# Small traumatic intracranial hemorrhages identified in routine radiology reports are associated with a low risk of adverse events: a retrospective cohort study

**DOI:** 10.1007/s00068-026-03199-0

**Published:** 2026-05-08

**Authors:** Karin Gerdås, Antonia Wigermo, Maja Offenbartl, Sebastian Vestlund, Francis Rezk, Tomas Vedin

**Affiliations:** 1 Department of Surgery, Högland Hospital, Region Jönköping county, Eksjö, Sweden; 2 Department of Surgery, Kungälv Hospital, Region Västra Götaland County, Kungälv, Sweden; 3 Department of Anesthesia and Intensive Care, Kalmar County Hospital, Kalmar, Sweden; 4 Lund University, Malmö, Sweden; 5 Department of Surgery, Ryhov Hospital, Region Jönköping County, Jönköping, Sweden; 6 Skåne University Hospital, Malmö, Sweden

**Keywords:** Traumatic intracranial hemorrhage, Neurosurgical intervention, Risk stratification, Radiology reports, Brain injury guidelines

## Abstract

**Purpose:**

To determine whether routinely available radiology reports, together with basic clinical data, can identify patients with traumatic intracranial hemorrhage (TICH) who are at low risk of adverse events.

**Methods:**

This retrospective cohort study of adults with TICH in Region Jönköping, Sweden (2019–2021). Clinical data, findings from radiology reports and outcomes were extracted from medical records. Hemorrhage size was classified as small (≤ 4 mm or described as minimal/very small/discrete) or larger. Adverse events were defined as neurosurgical intervention or death directly attributable to the TICH. Risk difference (RD), relative risk (RR), and Firth’s penalized logistic regression were used to assess associations with adverse events.

**Results:**

Among 527 included patients, 195 (37%) had small TICHs. None of these patients experienced adverse events, compared with 13.6% neurosurgical interventions and 13.0% trauma-related deaths in the group with larger TICHs (RD 24.5% points, 95% CI 18.4–29.6; RR 97.1, 95% CI 6.1–1557.1; *p* < 0.001). Small TICH size had the strongest association with absence of adverse events. Normal neurological status and GCS 14–15 were also associated with a low risk of adverse events. Anticoagulant or antiplatelet therapy showed no significant association with adverse events.

**Conclusion:**

Routinely available radiology reports, combined with basic clinical data, can identify a low-risk subgroup of patients with small TICHs. Hemorrhage size appears to be a useful factor for risk stratification, but the findings require internal and external prospective validation before implementation in clinical practice.

**Supplementary Information:**

The online version contains supplementary material available at 10.1007/s00068-026-03199-0.

## Introduction

Traumatic brain injury (TBI) is a common diagnosis in emergency departments (EDs), with an estimation of 12 deaths and 300 hospital admissions per 100 000 persons per year in Europe, including both children and adults [1, 2]. The majority of the TBIs are classified as mild, commonly defined as a Glasgow Coma Scale (GCS) score of 13–15 [3, 4]. Approximately 6.3–11% of the patients with mild TBI will have a traumatic intracranial hemorrhage (TICH) on computed tomography (CT), of which around 3.5% will require neurosurgical intervention and 1.4% will die due to the injuries [5–7].

Falls, either from ground-level or from elevation, represent the most frequent trauma mechanism causing TICH in high-income countries and among older adults, whereas motor vehicle accidents dominate in low- and middle-income countries and among younger individuals [8–14]. The incidence of TICH increases markedly with age, driven by higher prevalence of ground-level falls and the widespread use of antithrombotic medication [15]. Several factors have been associated with both the occurrence and progression of TICH, including advanced age, anticoagulant and antiplatelet therapy, alcohol intoxication, and comorbid conditions such as cardiovascular or neurological disease [12]. These factors increase both the likelihood of a sustaining TICH and the risk of adverse outcomes. Nevertheless, most hemorrhages following mild trauma remain small and clinically stable, and only a limited proportion of patients deteriorate or require neurosurgical intervention [16, 17].

The increasing use of antithrombotic therapy, mostly in the elderly population, poses an additional challenge in TICH management. Pre-injury anticoagulant use has been associated with increased mortality and need for neurosurgical intervention [18, 19]. Likewise, antiplatelet therapy has been linked to an elevated risk of both sustaining and worsening traumatic intracranial hemorrhage [17, 20] Yet, recent evidence suggest that in patients with small or clinically mild hemorrhages, anticoagulant or antiplatelet therapy does not appear to increase the risk of deterioration or adverse outcomes [16, 21].

Current management of TICH typically includes an initial head CT, neurosurgical consultation and often repeat imaging to monitor for hemorrhage progression. Depending on the extent of the injury, patients are either referred for neurosurgical care or admitted for in-hospital observation for at least 24 h [22, 23]. However, only a minority of the patients with GCS 13–15 deteriorate or require neurosurgical intervention [5]. Two recent Swedish studies have shown that 11.8% of patients with TICH needed acute neurosurgical intervention or died as a result of their intracranial injuries [24, 25]. Hence, it is conceivable that managing all patients with equal vigilance may be unnecessarily cautious and resource-intensive.

To address this, the Brain Injury Guidelines (BIG) from the United States [26] and the mild Traumatic Brain Injury Risk Score (mTBI RS) from the United Kingdom [27] were developed to stratify patients with CT-verified TICH according to injury severity and management needs, thereby identifying those at low risk of clinical deterioration or need for intervention. Both internal and external validation studies have confirmed that it may be safe to discharge patient in the lower-risk categories (BIG 1–2) without repeat CT scans or neurological consultation [27–29]. Khan et al. proposed modifications to the BIG criteria (mBIG) to further enhance patient safety, classifying all epidural hemorrhages as BIG 3, and also adding direct oral anticoagulants (DOACs) to BIG 3 alongside aspirin, warfarin and clopidogrel [23].

Marincowitz et al. developed mTBI RS, which also demonstrated a sensitivity of 99.5% for detecting patients at risk of deterioration and need for intervention. When tested on the same cohort, they found that the BIG criteria would discharge fewer patients, although this study cohort was older and had a lower GCS score [27]. However, it has previously been shown that neither the BIG nor the mTBI RS can be directly applied to Swedish clinical settings, as routine CT reports often lack the details required for their use [16].

The present study therefore aimed to determine whether routinely available radiology reports, in combination with basic patient characteristics, are sufficient to identify patients with traumatic intracranial hemorrhage at low risk of adverse events.

## Material and methods

This was a retrospective cohort study of medical records for patients diagnosed with TICH at Region Jönköping in Sweden between 2019 and 2021, encompassing one county hospital and two rural hospitals, serving a population of approximately 370 000 inhabitants. Patients requiring neurosurgical care are routinely transferred to Linköping University Hospital, the regional tertiary neurosurgical centre (approximately 120 km distance).

The inclusion criteria were:

- Age ≥ 18 years.
- Traumatic intracranial hemorrhage identified on the initial head CT scan.
- Admission to one of the three hospitals in Region Jönköping during the study period (2019–2021).

Patients were excluded if any of the following criteria were applied:

- Absence of initial head CT scan or missing medical records.
- Penetrating head injury.
- Initial management at a hospital outside Region Jönköping County.
- Transfer to another region during hospital stay (except for referral to the neurosurgical centre at Linköping University Hospital).

Potential cases were identified using International Classification of Diseases (ICD)-10 codes for traumatic head injuries for data extraction. Data were extracted manually by two independent reviewers using a predefined pro forma document (see appendix I) and guidelines for retrospective review by Vassar and Holzman were followed [30]. To minimize variability in interpretation, particularly of radiology report terminology, the reviewers conducted regular consensus meetings and discussions. All variable definitions and coding procedures were determined prior to data collection to ensure consistency. Extracted variables included information such as age, sex, comorbidities, mechanism of injury, medication (anticoagulant and antiplatelet therapy), level of consciousness, CT findings and clinical outcomes.

To enhance international applicability, the level of consciousness was reported throughout the study as the GCS. In Swedish clinical practice, hospitals routinely use the Reaction Level Scale (RLS-85) to assess the level of consciousness [31]. The RLS score was obtained from medical records or, when not explicitly stated, interpreted from the clinical descriptive documentation (e.g., “patient is awake and alert” corresponding to RLS = 1). It has previously been shown that RLS correctly classifies GCS 15 and 14, but discrepancies exist when converting RLS 3–8 to GCS 13 − 3. Therefore, level of consciousness was converted to GCS but to minimize bias, it was reported only in two categories: GCS 14–15 and GCS < 14 [32]. Normal neurological status was defined as the absence of new focal neurological deficits, preserved orientation and mental status, and no documented signs of neurological deterioration on arrival.

Information from radiology reports of the initial head CT was extracted and reviewed for all included patients. Medical records, including CT images, from the tertiary centre were made available for review in accordance with the ethical approval and locally applicable laws. Extracted variables comprised hemorrhage types, multiplicity, presence of skull and basilar fractures, evidence of previous hemorrhage, and reported size measurement when available. TICHs were classified as small if the reported size was ≤ 4 mm (according to the BIG) or < 5 mm (according to the mTBI RS). In cases where no numerical size was provided in the radiology report, bleedings described in the report using terms such as “minimal”, “very small” or “discrete” were also categorized as small. For risk-stratification purposes and in keeping with the criteria of both BIG and mTBI RS, the cohort was subsequently divided into low- and high-risk groups, using TICH size of ≤ 4 mm as the distinguishing threshold [26, 27].

Missing data in all parameters were coded explicitly but handled pragmatically by interpreting the absence of documentation as the absence of pathology (e.g., no mention of loss of consciousness was interpreted as “no loss of consciousness”). This approach reflects routine clinical practice, where positive findings are typically documented while normal or absent findings may not be stated. Similar handling of undocumented findings has been applied in previous regional TICH studies [16, 17, 33], and was therefore considered to involve an acceptable risk of systematic bias given that the extracted parameters are routinely assessed in the management of TBI patients.

Adverse events were defined as any neurosurgical intervention (craniotomy, trepanation, or intracranial pressure monitoring) or death directly related to the TICH (e.g., neurological deterioration following the head trauma with worsening level of consciousness and ultimately death due to brain herniation).

The primary outcome measures were risk difference (RD) and relative risk (RR) between the subgroups of patients with small and larger TICHs, as described above. The secondary outcome measure was the Odds Ratio (OR) for the association between clinical and radiological characteristics and the occurrence of adverse events.

### Ethical considerations

This study was approved by the Swedish Ethical Review Authority (Dnr 2022-05681-01). All data were pseudonymized prior to analysis, and the study was conducted in accordance with the Declaration of Helsinki and applicable national data protection legislation (including the General Data Protection Regulation). As the data were securely stored and pseudonymized, with minimal risk of patient identification, both the investigators and the Ethical Review Authority considered the risk of patient harm to be low. Consequently, the requirement for informed consent was waived.

### Statistical analysis

Data were analysed using IBM SPSS Statistics v 29. The distribution of continuous variables was assessed using histograms and the Shapiro-Wilk test to determine whether data were normally distributed. Continuous variables were summarized as medians with interquartile ranges (IQR) or means with standard deviations (SD), as appropriate. Group comparisons were performed using Fisher’s exact test for categorical variables.

Risk differences (RD) with 95% confidence intervals (CI) were calculated using the Newcombe–Wilson method without continuity correction, which provides accurate interval estimates even when one group has zero events [34]. Relative risks (RR) with corresponding 95% CIs were calculated using the Haldane-Anscombe correction, where 0.5 was added to each cell when one or more cells contained zero counts [35]. To assess associations between clinical and radiological variables and adverse events, Firth’s penalized logistic regression was subsequently applied to reduce small-sample bias and address complete or quasi-complete data separation. Results were presented as odds ratios (OR) with 95% confidence intervals (CI) and corresponding p-values. A p-value < 0.05 was considered statistically significant.

## Results

**Fig. 1:**
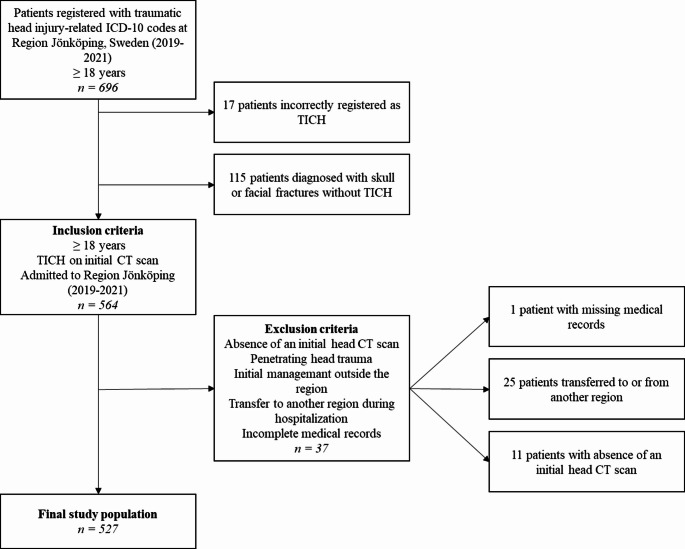
Inclusion flow chart. Inclusion flowchart of patient selection. A total of 696 patients (≥ 18 years) were registered with traumatic head injury-related ICD-10 codes in Region Jönköping, Sweden (2019–2021). These ICD-10 codes included TICH and fractures of the skull, skull base or facial bones. Of the 564 patients who met the inclusion criteria, 37 were excluded according to predefined exclusion criteria, resulting in a final study population of 527 patients. (TBI=traumatic brain injury; TICH=traumatic intracranial hemorrhages; CT=computed tomography; ICD-10 = International Classification of Diseases, 10th revision)

A total of 696 medical records of patients with ICD-10 codes indicating TICH or fractures of the skull, skull base or facial bones were reviewed, of which 527 fulfilled the predefined inclusion criteria (Fig. 1). The median age of the study population was 76 years (interquartile range 62–86), with a slight male predominance. The most common mechanisms of injury were falls accounting for 79% (61% same-level falls and 18% falls from height), followed by motor vehicle accidents (12%) and other mechanisms (each ≤ 4%) (Table 1).

**Table 1:** Patient characteristics and outcomes of the study population, stratified by hemorrhage size

Variable name	Variable sub-category	All TICHs	Small TICHs	Larger TICHs
Number of patients	n (%)	527 (100.0)	195 (37.0)	332 (63.0)
Age				
	Median (Q1-Q3) years	76 (62–86)	72 (58–83)	79 (69–85)
Gender				
	Female (%)	241 (45.7)	89 (45.6)	152 (45.8)
	Male (%)	286 (54.3)	106 (54.4)	180 (54.2)
Comorbidity*	Any comorbidity (%)	221 (41.9)	100 (51.3)	206 (62.0)
Antithrombotic treatment				
	Anticoagulant (%)	106 (20.1)	36 (18.5)	70 (21.1)
	Antiplatelet (%)	149 (28.3)	40 (20.5)	109 (32.8)
	Dual antiplatelets (%)	11 (2.1)	4 (2.1)	7 (2.1)
Trauma mechanism				
	Ground-level fall (%)	320 (60.7)	111 (56.9)	209 (63.0)
	Elevated fall (%)	96 (18.2)	39 (20.0)	57 (17.2)
	Motor vehicle accident (%)	62 (11.8)	26 (13.3)	36 (10.8)
	Assault (%)	9 (1.7)	4 (2.1)	5 (1.5)
	Sport activity (%)	8 (1.5)	5 (2.6)	3 (0.9)
	Other (%)	11 (2.1)	5 (2.6)	6 (1.8)
	Unknown (%)	21 (4.0)	5 (2.1)	16 (4.8)
Time from trauma to arrival				
	< 24 h (%)	479 (90.9)	182 (93.3)	297 (89.5)
	> 24 h (%)	44 (8.3)	11 (85.6)	33 (9.9)
	Unknown (%)	4 (0.8)	2 (1.0)	2 (0.6)
Clinical findings upon arrival				
	Level of consciousness			
	GCS 14–15 (%)	479 (90.9)	192 (98.5)	287 (86.4)
	GCS < 14 (%)	48 (9.1)	3 (1.5)	45 (13.6)
	Loss of consciousness (%)	319 (60.5)	114 (58.5)	205 (61.7)
	New focal neurological deficits (%)	68 (12.9)	9 (4.6)	59 (17.8)
	Intoxication (%)	89 (16.9)	41 (21.0)	48 (14.5)
Days admitted	Median (Q1-Q3)	3 (1–6)	2 (1–4)	3 (1–7)
CT scans during hospital stay	Median (Q1-Q3) number of CT scans	2 (2–3)	2 (2–3)	2 (1–3)
Outcomes				
	Neurosurgical intervention (%)	45 (8.5)	0 (0)	45 (13.3)
	Intensive unit care (%)	92 (17.5)	10 (5.1)	82 (24.7)
	Clinical deterioration (%)	64 (12.1)	3 (1.5)	61 (18.4)
	Neurosurgical consultation (%)	505 (95.8)	181 (92.8)	324 (97.6)
	Death			
	within 24 h (%)	13 (2.5)	0 (0)	13 (3.9)
	within 30d (%)	60 (11.4)	2 (1.0)	58 (17.5)
	directly caused by the TICH (%)	43 (8.2)	0 (0)	43 (13.0)
	Readmittance 30d (%)	26 (4.9)	0 (0)	26 (7.8)

Values are presented as numbers with percentages unless otherwise specified. Continuous variables are expressed as median values with interquartile range (Q1–Q3). “Small TICHs” were defined as hemorrhages ≤ 4 mm or described as minimal, very small, or discrete in the radiology report. “Larger TICHs” were defined as hemorrhages > 5 mm or described as large, significant or extensive bleedings in the radiology report

^a^Any comorbidity was defined as the presence of any of the following cardiovascular, pulmonary, renal and neurological disease, diabetes mellitus or dementia

(TICH = traumatic intracranial hemorrhage; CT = computed tomography; GCS = Glasgow Coma Scale; ICU = intensive care unit)

As summarized in Table 2, acute subdural hemorrhage was the most frequent type of intracranial bleedings, followed by subarachnoid hemorrhage. Multiple hemorrhage types occurred in 33% of the patients. Almost 10% had concomitant basilar skull fractures, 8% had other skull fractures, and 8% had facial fractures.

Sixty patients (11.4%) died after the initial TICH, of which 43 deaths (8.2%) were considered directly attributable to the TICH. Neurosurgical intervention was performed in 45 patients (8.5%), all of whom belonged in the high-risk group with larger TICHs. Twenty-five patients (4.7%) received antibiotic treatment due to skull fractures, and, in total, 92 (17.5%) were admitted to intensive care unit (ICU) for observation or monitoring, irrespective of the underlying cause.

**Table 2:** Radiological findings and hemorrhage size

Radiological findings (*n* = 527)		*n* (%)
TICH types		
	Multiple different hemorrhages (%)	173 (32.8)
	Acute subdural hemorrhage (%)	184 (34.9)
	Chronic subdural hemorrhage (%)	5 (0.9)
	Epidural hemorrhage (%)	4 (0.8)
	Subarachnoidal hemorrhage (%)	98 (18.6)
	Parenchymal hemorrhage (%)	28 (5.3)
	Contusion hemorrhage (%)	18 (12.3)
	Other intracranial hemorrhage (%)	3 (0.6)
	Hemorrhage with uncertain localization	14 (2.7)
Secondary radiological signs		
	Midline shift	98 (18.6)
Skull and facial fracture(s)		
	Basilar skull fracture (%)	50 (9.5)
	Other skull fracture/s (%)	41 (7.8)
	Facial fracture/s (%)	44 (8.3)
TICH size		
	Small size of TICH	195 (37.0)
	specified	69 (35.4)
	non-specified^a^	126 (64.3)
	Larger size of TICH	332 (63.0)
	specified	292 (88.0)
	non-specified^a^	40 (12.0)

The size of traumatic intracranial hemorrhage (TICH) was either classified as specified in the radiology report or non-specified. Non-specified^a^ small TICHs were defined based on descriptive terms such as “minimal, very small or discrete bleeding”, while larger TICHs were described as “large, significant or extensive bleeding”

Of the 195 patients in the low-risk group, the median age was 72 years and the median hospital stay was two days. Each patient underwent a median of two head CT scans (one at the emergency room and one during the in-patient stay) and a neurosurgical consult was made in 92.8% of cases. Anticoagulant therapy was used by 36 patients (18.5%), antiplatelet therapy by 40 (20.5%), and 4 (2.1%) received dual antiplatelet therapy.

Two elderly patients (88 and 91 years old) died within 30 days after the head trauma. Both were re-admitted with infectious symptoms, and follow-up CT scans showed partial or complete regression of the previous hemorrhage (deaths occurred 25 and 30 days after injury, respectively) and were therefore considered unrelated to the TICH. Clinical deterioration was observed among three patients during hospitalization (one with seizure with known epilepsy and two with transient altered consciousness), but none required neurosurgical intervention or showed progression on follow-up CT.

None of the patients in the low-risk group had adverse events, compared with 13.6% undergoing neurosurgical intervention and 13.0% dying from the TICH, respectively, in the high-risk group. Taken together, this corresponded to a RD of 24.5% points (95% CI 18.4–29.6) and a RR of 97.1 (95% CI 6.1-1557.1; *p* < 0.001) for adverse events among patients with larger TICHs.

In the regression analysis, small TICH size was independently associated with the absence of adverse events and had the highest OR of all variables included in the regression (OR 86.49, 95% CI 12.16-10 970.88, *p* < 0.001). Normal neurological status upon arrival to the hospital (OR 5.75, 95% CI 2.82–12.97. *p* = 0.047) and a GCS 14–15 (OR 3.56, 95% CI 1.56–8.17, *p* = 0.003) were also significantly associated with the absence of adverse events. Male sex was inversely associated with the outcome (OR 0.39, 95% CI 0.21–0.71, *p* = 0.002), whereas age, anticoagulant or antiplatelet therapy, intoxication, loss of consciousness and comorbidity showed no significant associations (Table 3).

**Table 3:** Firth’s penalized logistic regression model assessing factors associated with no adverse events

Variable	OR	95% CI	*P*-value
Age (over 65 years)	0.88	(0.13–1.21)	0.758
Anticoagulant therapy	0.89	(0.37–2.03)	0.772
Antiplatelet therapy	1.19	(0.41–2.03)	0.651
Loss of consciousness	1.48	(0.81–2.75)	0.205
Sex (male)	0.39	(0.21–0.71)	0.002
Any comorbidity^a^	0.85	(0.40–1.80)	0.679
Normal neurological status	5.75	(2.82–11.97)	< 0.001
Intoxication	2.14	(0.86–5.96)	0.105
GCS 14–15	3.56	(1.56–8.17)	0.003
Small size of TICH	86.49	(12.16-10 970.88)	< 0.001

No adverse events were defined as no neurosurgical intervention or death attributable to the traumatic intracranial hemorrhage (TICH)

(OR=Odds ratio; CI=Confidence interval; GCS=Glasgow coma scale)

^a^Any comorbidity was defined as the presence of any of the following cardiovascular, pulmonary, renal and neurological disease, diabetes mellitus or dementia

## Discussion

This study demonstrates that routinely available radiology reports, combined with basic clinical variables, can be used to identify patients with TICH at low risk of adverse events. Patients with small TICHs had a favorable clinical course, with no neurosurgical interventions or deaths directly attributable to the TICH. The risk for adverse events was significantly lower in the low-risk group, and regression analysis further supported this finding, showing that a small TICH size was most strongly associated with the absence of adverse events.

The benign course of small TICHs may likely reflect negligible mass effect and minimal disruption of surrounding brain tissue, resulting in lower risk of secondary bleeding expansion and intracranial pressure elevation [36, 37]. Normal neurological status and a GCS of 14–15 on admission were also significantly associated with the absence of adverse events. The association with normal neurological status is consistent with previous studies indicating that neurological injury, rather than initial CT findings alone, is the most reliable indicator of poor outcome [38]. The association with a GCS of 14–15 likely reflects that these patients presented with less severe TICH and preserved neurological function. Male gender, however, was inversely associated with the outcome, indicating that female patients would be more likely to have a favorable course without adverse events.

Our results aligned closely with previous international validation studies of the BIG and mTBI RS, which show that only a small proportion of patients with TICH required neurosurgical intervention (8.5%) or died due to the hemorrhage (8.2%, slightly higher than in the previous studies) [23, 27, 29]. Patients with small TICHs in our cohort showed no neurosurgical interventions or deaths directly caused by the hemorrhage, corresponding closely to the lowest-risk categories in both the BIG (BIG 1) and mTBI RS models. This lends further support to the validity of our findings and suggests that the outcomes observed in our cohort are comparable to those reported internationally.

Notably, while the BIG and mTBI RS classify patients receiving anticoagulant or antiplatelet therapy into higher-risk groups, our findings indicate that the presence of such medication did not translate into an increased risk of adverse events when the hemorrhage was small. Together, these findings support simplified risk stratification models for TICH. However, unlike the BIG and mTBI RS, which rely on detailed CT measurements, our results suggest that routinely available radiology reports might point toward a similar low-risk group, although direct comparisons are limited. In clinical practice, reliance on descriptive terminology rather than precise measurement may limit confidence in management decisions.

Khan et al. (2022) demonstrated that implementing the mBIG could substantially reduce the number of repeat CT scans and neurosurgical consultations without increasing the risk of missed interventions or mortality [39]. In our cohort, nearly all patients with small TICHs underwent repeat imaging and received neurosurgical consultation as part of routine surveillance to confirm radiological stability, despite the absence of adverse events, suggesting that the use of repeat imaging and consultation may be reduced in selected low-risk patients.

Importantly, neither pre-injury anticoagulant nor antiplatelet therapy was significantly associated with adverse events. Among the patients with small TICHs, nearly 40% were receiving either anticoagulant or antiplatelet treatment, yet none required neurosurgical intervention or died as a result of the hemorrhage. These findings indicate that, in the context of small TICHs, the use of medications affecting coagulation does not appear to increase the risk of adverse events. This supports recent evidence that, in patients with small or clinically mild hemorrhages, antithrombotic therapy does not necessarily increase the risk of progression or clinical deterioration [12, 15, 29].

Previous studies have shown that pre-injury anticoagulant and antiplatelet therapy are associated with poorer outcomes following TICH, including higher mortality and increased rates of neurosurgical intervention [17–19]. Warfarin appears to confer a greater risk than DOACs [21], while antiplatelet therapy has been linked to higher as well as lower risk compared with both warfarin and DOACs [17, 20]. However, when the hemorrhages are small, as in the present study, these associations seem to be of limited clinical relevance. In contrast to these earlier studies, our analysis took hemorrhage size into account, which may explain the lack of association between antithrombotic therapy and adverse events. It is possible that the increased risk observed in earlier cohorts was primarily related to patients with larger or progressing hemorrhages.

The cohort showed a similar age distribution, prevalence of antithrombotic therapy and mechanism of injury (predominantly falls) as previously reported in Scandinavian and other European studies [17, 27, 40]. In contrast, the American BIG cohort described by Joseph et al. [26, 29] included a younger study population with fewer patients on antithrombotic therapy and a predominance of motor vehicle accidents rather than falls, and comparisons with our cohort should therefore be interpreted with caution. Nevertheless, the consistency of our findings with European data suggests that the observed patterns may reflect typical TICH populations encountered in comparable healthcare contexts.

Our findings suggests that a substantial proportion of patients with TICH may not require acute intervention and could potentially be managed with early discharge when clinically stable. Identifying this low-risk group may improve resource efficiency and limit exposure to risks associated with hospitalization, while maintaining patient safety. However, the reliance on descriptive terminology in routine CT reports introduces subjectivity, which limits the immediate clinical applicability of these findings.

Several limitations of the present study should be acknowledged. It was retrospective in design and based on routinely documented clinical data, which may introduce bias. In particular, radiology reports often lacked precise measurements of hemorrhage size, requiring classification based on descriptive terminology rather than quantified dimensions. As CT scans were interpreted as part of routine clinical care across multiple hospitals without standardized definitions for descriptive hemorrhage size terminology, interobserver variability between radiologists may have influenced classification, thereby limiting the applicability of these findings. Nevertheless, as this approach reflects routine clinical practice, the findings may still be clinically relevant, although standardized reporting would be required for consistent implementation.

Moreover, missing data were pragmatically interpreted as absence of pathology, which, although reflecting assumed typical documentation patterns, may have introduced systematic bias. Furthermore, the low number of adverse events limited statistical power. Although Firth’s penalized regression was applied to reduce small-sample bias, the resulting wide confidence intervals likely reflect the rarity of adverse events, with no adverse events in the small TICH group resulting in large odds ratios that should be interpreted with caution.

## Conclusion

This regional cohort suggests that routinely available radiology reports, combined with basic clinical data, can identify a low-risk subgroup of patients with small TICHs. Hemorrhage size appears to be a useful factor for risk stratification, but the findings require internal and external prospective validation before implementation in clinical practice.

## Electronic Supplementary Material

Below is the link to the electronic supplementary material.

Supplementary Material 1

## Data Availability

The datasets generated and analyzed during the current study are not publicly available due to Swedish confidentiality regulations but are available from the corresponding author on reasonable request.
