# Supplementary material for: Small traumatic intracranial hemorrhages identified in routine radiology reports are associated with a low risk of adverse events: a retrospective cohort study
Source: Eur J Trauma Emerg Surg. 2026 May 8;52(1):160. doi: 10.1007/s00068-026-03199-0 (PMC13156081; doi:10.1007/s00068-026-03199-0)
Supplement: Supplementary file 1 — Supplementary Material 1 [file 68_2026_3199_MOESM1_ESM.docx]

# Appendices

## Appendix I

Pro forma document, Coding Manual

#### 1. SEX – Sex

0 = Female
1 = Male

#### 2. AGE – Age

Completed years

#### 3. n_DAYS – Number of days in hospital

Number of full hospital days

#### 4. n_HOURS – If less than one day, number of hours

HH:MM
88 = Not applicable

#### 5. EXKL – Excluded?

0 = No
1 = Yes

#### 6. EXKL_WHY – If excluded, specify reason

0 = Not excluded
1 = Incorrectly registered as intracranial injury or non-traumatic lesion
2 = Duplicate
3 = Readmission for an already included injury
4 = Transferred from another hospital
5 = Record with restricted confidentiality
6 = Transferred to another region before discharge
7 = Deceased after the most recent registered admission
8 = Missing initial CT scan
9 = Patient from another region / foreign resident
10 = Penetrating injury, including gunshot wound
11 = Only facial fracture without intracranial injury/skull fracture
12 = Insufficient information for inclusion

#### 7. TIME_TRAUMA – Time from trauma to emergency department presentation

1 = 0–6 hours
2 = 6–12 hours
3 = 12–24 hours
4 = <24 hours (unspecified within the first day)
5 = >24 hours
6 = >1 week
7 = >1 month
99 = Missing data

If the time cannot be precisely determined, mark 99.
If it is clear that the trauma occurred within 24 hours but cannot be specified further, use option 4.

#### 8. MECHANISM – Mechanism of trauma

1 = Ground-level fall (from standing or sitting)
2 = Fall from height
3 = Assault
4 = Traffic accident
5 = Sports injury
6 = Other
7 = Unknown
99 = Not stated in the medical record

#### 9. AMNESIA – Post-traumatic amnesia

0 = No
1 = Yes
2 = Not mentioned in the record

#### 10. LOC – Loss of consciousness

0 = No
1 = Yes
2 = Not mentioned in the record
3 = Uncertain/possible
99 = Missing data

#### 11. LOC_DURATION – Duration of loss of consciousness (if applicable)

1 = “Momentary”
2 = <1 minute
3 = >1 minute but <5 minutes
4 = >5 minutes
5 = Patient cannot recall
6 = Unconscious on arrival
88 = Not applicable
99 = Missing data or not specified

#### 12. INTOX – Intoxication

0 = Not intoxicated
1 = Alcohol
2 = Cannabis
3 = Central stimulants (Cocaine, Amphetamine, Methamphetamine, Methylphenidate, LSD, Ecstasy)
4 = Opiates (Heroin, Morphine, Oxycodone, Codeine, Tramadol)
5 = Benzodiazepines (Alprazolam, Clonazepam, Diazepam, Oxazepam)
6 = Unknown substance
7 = Patient unable to specify
8 = Other substance
9 = Mixed intoxication
99 = Missing data

Any note of substance use, positive lab result, smell of alcohol, or other indication is coded as intoxicated.
“Unknown substance” is used when the type of drug is unspecified.
“Not intoxicated” is used if the record explicitly states absence of intoxication.

#### 13. SEIZURE – Seizures

0 = No
1 = Yes, unobserved
2 = Yes, observed
3 = Not mentioned in the record
4 = Possible seizure
99 = Missing data

#### 14. VOMIT_PREHOSP – Vomiting (prehospital)

0 = No
1 = Yes
99 = Not mentioned in the record

#### 15. n_VOMIT_PREHOSP – Number of vomiting episodes

Numeric entry
88 = Not applicable
99 = Can not be determined with certainty

#### 16. HEADACHE – Headache after trauma

0 = No
1 = Yes
99 = Not mentioned in the record

#### 17. DOC_RLS – Formal documentation of RLS (Reaction Level Scale) in ED record

0 = No
1 = Yes

#### 18. DOC_RLS_LEVEL – If yes, RLS level at first assessment

1–8
88 = Not applicable

#### 19. INT_RLS – If no formal RLS documented, can it be inferred from record?

0 = No
1 = Yes

#### 20. INT_RLS_LEVEL – If yes, inferred RLS level

1–8
88 = Not applicable

#### 21. NORM_NEURO_EX – Normal neurological examination on admission

0 = No (abnormal findings)
1 = Yes (normal neurological exam)
2 = Cannot be assessed due to insufficient documentation
3 = No neurological examination documented

#### 22. EQ_PUPILS – Pupils equal in size at first examination

0 = No
1 = Yes (normal)
2 = Not mentioned in the record
3 = Not assessable (e.g., one eye swollen shut, enucleated eye, etc.)

#### 23. REACT_PUPILS – Pupillary light reactivity at first examination

0 = No
1 = Yes (normal)
2 = Not mentioned in the record
3 = Not assessable

#### 24. SIGNS_SKULLFX – Clinical signs of skull fracture

0 = No signs of skull fracture
1 = Palpable step or depression
2 = Palpable fluctuation of cranial bone
3 = Periorbital hematoma (raccoon eyes)
4 = Retroauricular hematoma (Battle’s sign)
5 = Hemotympanum
6 = Clear fluid from ear or nose (CSF leakage)
7 = Multiple signs present
88 = Not mentioned in the record

#### 25. COAGPATH – Known congenital coagulation disorder

0 = No
1 = Yes

If not mentioned in the record, code as 0.

#### 26. CARDVASC_DIS – Cardiovascular disease

0 = No
1 = Yes
Includes:

- Previous myocardial infarction
- Heart failure
- Previous TIA or stroke
- Peripheral vascular disease
- History of revascularization (CABG, PCI, PTA)
- Heart transplantation

#### 27. DIABETES_MELL – Diabetes mellitus

0 = No
1 = Yes

#### 28. PULM_DIS – Pulmonary disease

0 = No
1 = Yes
Includes:

- COPD
- Pulmonary fibrosis
- Severe asthma

#### 29. ESRD – End-stage renal disease (on dialysis) or chronic renal failure with eGFR <30

0 = No
1 = Yes

#### 30. LIVER_DIS – Liver disease

0 = No
1 = Yes
Includes:

- Liver cirrhosis
- Other chronic liver disease with elevated transaminases

#### 31. NEURO_DIS – Neurological disease

0 = No
1 = Yes
Includes:

- Multiple sclerosis
- Amyotrophic lateral sclerosis
- Epilepsy
- Guillain–Barré syndrome
- Myasthenia gravis
- Parkinson’s disease
- Brain tumor

#### 32. DEMENTIA – Dementia

0 = No
1 = Yes

Regardless of cause or severity.

#### 33. PSYCH_DIS – Psychiatric disease

0 = No
1 = Yes
Defined as:

- Severe depression
- Bipolar disorder
- Chronic psychotic disorders
- Dementia with delusions requiring inpatient psychiatric care within the past 5 years

#### 34. ANTICOAG – Anticoagulant therapy

0 = No
1 = Warfarin
2 = Apixaban (Eliquis)
3 = Dabigatran (Pradaxa)
4 = Rivaroxaban (Xarelto)
5 = Edoxaban (Lixiana)
6 = Low molecular weight heparin (LMWH)
7 = Other
99 = Data missing

If “current medication” is listed in the record but no anticoagulant is mentioned, code as 0.
Also code 0 if medication list is missing but the patient has no known indication for anticoagulation.
If the indication is present but the medication is not specified, code as 99.

#### 35. TRCINHIB – Antiplatelet therapy

0 = No
1 = Acetylsalicylic acid (ASA)
2 = Clopidogrel (Plavix)
3 = Ticagrelor (Brilique)
4 = Prasugrel (Efient)
5 = Dipyridamole (Persantin)
6 = Cilostazol (Pletal)
99 = Data missing

#### 36. DBLTRCINHIB – Dual antiplatelet therapy

Define as presence of a second antiplatelet agent listed under point 35.

#### 37. DEATH_30D – Death within 30 days from initial hospital visit

0 = No
1 = Yes

Calculated as 30 days from discharge date. Applies also to other 30-day measures.

#### 38. DEATH_ADM – Death during index hospital admission

0 = No
1 = Yes

#### 39. DEATH_24H – Death within 24 hours of admission

0 = No
1 = Yes

#### 40. DEATH_ICI – Death secondary to traumatic intracranial injury

0 = No
1 = Yes
88 = Not applicable

#### 41. DEATH_ICI_DoI – Direct or indirect cause of death from traumatic intracranial injury

1 = Direct
2 = Indirect
88 = Not applicable

#### 42. DEATH_DAYS_AFTER – Number of days from trauma to death

Numeric entry
88 = Not applicable

#### 43. NEURO_INT_30D – Neurosurgical intervention or ICU care due to head injury

0 = No
Yes defined as:
1 = Craniotomy
2 = Burr hole trepanation
3 = Intracranial pressure monitoring
4 = Mannitol treatment
5 = Hyperventilation therapy
6 = Hypertonic saline therapy
7 = Intravenous antibiotics for skull base fracture
8 = Other intervention
9 = Multiple interventions
10 = Observation in neuro-intensive care
11 = Angiography
12 = Operation for skull fracture
13 = Shunt adjustment
88 = Not applicable

#### 44. CLINICAL_DET – Clinical deterioration during hospital stay

0 = No
Yes defined as:
1 = Seizure or repeated seizures
2 = Decreased level of consciousness
3 = Neurological deterioration
4 = Newly developed or worsened confusion
5 = Marked worsening of headache
99 = Data missing

#### 45. READMIT_30D – Unplanned hospital readmission within 30 days related to the injury

0 = No
1 = Yes

#### 46. READMIT_DATE – Number of days from trauma to readmission

Numeric entry
88 = Not applicable

#### 47. CT_INJ_1 – Findings on initial head CT

1 = Acute subdural hemorrhage
2 = Subacute/chronic subdural hemorrhage
3 = Epidural hemorrhage
4 = Subarachnoid hemorrhage
5 = Parenchymal hemorrhage
6 = Contusion hemorrhage
7 = Other intracranial hemorrhage
8 = Signs of diffuse axonal injury (DAI)
9 = Cerebral edema
10 = Skull base fracture
11 = Comminuted fracture without depression
12 = Comminuted fracture with depression
13 = Linear fracture without depression
14 = Linear fracture with depression
15 = Other skull fracture
16 = Hemorrhage difficult to characterize
20 = Facial fracture(s) only
88 = No intracranial injury or fracture
99 = CT not performed or not available

#### 48. CT_INJ_2 – Findings on repeat CT

0 = Unchanged
1–16 = New findings (same coding as point 47)
17 = Progression of previous hemorrhage
18 = Regression of previous hemorrhage
19 = Increased midline shift
88 = Performed but result missing
99 = Not performed

#### 49. CT_INJ_3 – Findings on CT #3

Use same coding as point 47 and 48
99 = Not performed

#### 50. CT_INJ_4 – Findings on CT #4

Use same coding as point 47
99 = Not performed

#### 51. CT_INJ_5 – Findings on CT #5

Use same coding as point 47.
99 = Not performed

#### 52. n_CT_INJ – Number of different types of injuries on CT

Numeric entry

#### 53. OLD_CT_INJ – Evidence of previous intracranial injury on CT

0 = No
1 = Yes

Only previously diagnosed intracranial hemorrhages or fractures are coded here.
Signs of old infarction should **not** be coded as old intracranial injury.

#### 54. MID_SHIFT_WRITTEN – Midline shift or mass effect mentioned in radiology report

0 = No (no shift described)
1 = Yes (midline shift or mass effect present)
2 = Not mentioned in report

#### 55. MID_SHIFT_MM – If midline shift present, number of millimeters

Numeric entry
88 = Not applicable
99 = Measurement not specified in report

#### 56. LARGEST_BLEED_WRITTEN – Written comment on size of the largest hemorrhage

0 = No
1 = Yes

#### 57. LARGEST_BLEED_MM – If yes, size in millimeters

Numeric entry
0 = Present but no measurement provided
88 = Not applicable

#### 58. LT4MM_WRITTEN – Radiology report suggests hemorrhage <4 mm

0 = No
1 = Yes
88 = Not applicable (size already specified under point 57)

#### 59. REP_CT – At least one repeat head CT performed during hospital stay

0 = No
1 = Yes
88 = Not applicable
99 = Not mentioned in radiology report

#### 60. REP_CT_WHY – Reason for repeat CT

1 = Decreased level of consciousness
2 = Increasing headache
3 = New neurological abnormality
4 = Vomiting
5 = Other reason
6 = Reason not stated
7 = Complementary imaging
8 = New trauma event
88 = Not applicable

#### 61. CT_n – Total number of CT scans performed

Numeric entry

#### 62. MR_ – MRI performed

0 = No
1 = Yes

#### 63. MR_INJ – MRI findings

0 = No abnormality
1 = Diffuse axonal injury (DAI) grade 1
2 = DAI grade 2
3 = DAI grade 3
4 = Other MRI findings

#### 64. AIS_C-SPINE – Highest Abbreviated Injury Scale (AIS) score from head, neck or cervical spine

0–6 (use numeric value)
Definitions:
1 = Minor injury
2 = Moderate injury
3 = Serious injury
4 = Severe (life-threatening but likely survivable)
5 = Critical (survival uncertain)
6 = Maximal (unsurvivable)
9 = Unknown injury

Exclude superficial wounds, hematomas and abrasions (code as 0).
If multiple injuries within the same region, select the highest AIS score.

#### 65. AIS_F-FACIAL – Highest AIS score from face (including facial skeleton, nose, mouth, eyes and ears)

Use same definitions as in point 64.

#### 66. AIS_T-SPINE – Highest AIS score from thorax (including diaphragm and thoracic spine)

Use same definitions as in point 64.

#### 67. AIS_L-SPINE – Highest AIS score from abdomen (including internal pelvic organs and lumbar spine)

Use same definitions as in point 64.

#### 68. AIS_E-EXT – Highest AIS score from extremities and pelvic skeleton

Use same definitions as in point 64.

#### 69. AIS_S-SOFT_TISSUES – Highest AIS score from external soft tissues (skin, subcutaneous tissue)

Use same definitions as in point 64.

#### 70. VIT_PARA – Vital parameters on arrival

0 = Normal vital signs
1 = One abnormal vital sign
2 = Several abnormal vital signs
99 = Data missing

**Normal vital signs defined as:**

- Respiratory rate: 10–20 breaths/min
- SpO₂ > 95%
- Pulse: 50–100 beats/min
- Blood pressure: 100–140 / 60–90 mmHg

If the patient has a habitual deviation (e.g., chronic tachycardia), code based on deviation from their baseline.

#### 71. INT_CA – Intensive care unit admission

0 = No
1 = Yes

#### 72. INT_CA_days – Number of ICU days

N = days at ICU
88 = Not applicable
